# Tumefactive Demyelinating Lesions: An Illustrative Pediatric Case With an Atypical Presentation and Literature Review

**DOI:** 10.7759/cureus.61207

**Published:** 2024-05-28

**Authors:** Meryem Moro, Nissrine Louhab, Mohamed Chraa, Najib Kissani

**Affiliations:** 1 Neurology, Mohammed VI University Hospital of Marrakesh, Marrakesh, MAR

**Keywords:** tumefactive demyelinating lesions, tumefactive demyelination, neuromyelitis optica spectrum disorder (nmosd), mogad, multiple sclerosis and other demyelinating disorders

## Abstract

Tumefactive demyelinating lesions remain a rare entity and a source of diagnostic difficulty. Here, we report the case of a teenage girl who presented with a one-month history of progressive quadriparesis and symptoms of intracranial hypertension. Brain MRI showed multiple large subcortical white matter lesions with both open- and closed-rim enhancement on gadolinium injection. The patient subsequently underwent a brain biopsy which showed an inflammatory infiltrate and no signs of malignancy. She was treated with pulse intravenous methylprednisolone at a dose of 500mg per day for five days and had rapid improvement. Her symptoms fully resolved after three months. This case highlights the need for better recognition and diagnosis of tumefactive demyelination, potentially avoiding unnecessary invasive diagnostic procedures such as brain biopsies.

## Introduction

Tumefactive demyelinating lesions (TDLs), otherwise known as pseudotumoral or tumor-like demyelination, refers to large demyelinating lesions arbitrarily defined as exceeding 2 cm on T2-weighted brain MRI [1,2].

They can occur at any age, either as a solitary lesion or as multiple lesions [3-5]. They are most often associated with multiple sclerosis (MS) but can also be found in other acquired demyelinating diseases of the central nervous system (CNS) such as neuromyelitis optica spectrum disorder (NMOSD), myelin oligodendrocyte glycoprotein antibody-associated disease (MOGAD), acute disseminated encephalomyelitis (ADEM) and MS variants such as Balo’s concentric sclerosis, Schilder’s disease or Marburg’s acute multiple sclerosis [1].

We report the case of a teenage girl with an atypical presentation of progressive quadriparesis and intracranial hypertension evolving over one month, revealing TDLs.

## Case presentation

A 14-year-old girl with no prior medical history presented to the neurology department with progressive quadriparesis occurring over one month. She first developed lower limb weakness: beginning with the right lower limb followed three days later by the left one. Two weeks later, she developed slight upper limb weakness with the same pattern of progression: first the right, then the left upper limb.

She reported very mild left facial weakness only when prompted. In addition, she started to experience new-onset diffuse headaches with progressive worsening, nausea, and vomiting. The day before admittance, she developed horizontal diplopia.

On examination, she had spastic quadriplegia with brisk deep tendon reflexes, Babinski signs, and mild left central facial weakness. She also had incomplete bilateral VIth nerve palsy. Ophthalmologic exam including visual acuity was normal.

**Figure 1:**
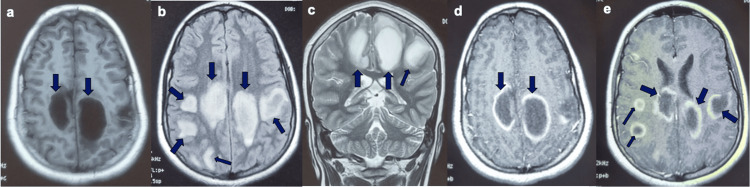
Brain MRI: (a) T1 axial, (b) FLAIR axial (c) T2 coronal, (d) and (e) T1 axial with gadolinium injection

A brain MRI showed multiple bilateral large subcortical white matter T1 hypointensities and T2/FLAIR hyperintensities, the most voluminous measuring 29×38 mm, with intense closed and open ring enhancement after gadolinium injection (Figure 1). Spinal cord MRI was without abnormalities.

Further workup included laboratory investigations, with complete blood count (CBC), metabolic profile, and infectious screening, all of which were normal. Cerebrospinal fluid (CSF) was obtained and proved to be unremarkable with normal glucose, protein, and cell count. Chest, abdomen, and pelvis CT were also obtained to rule out a possible malignant process with metastases. All were negative.

At this point, the patient underwent a brain biopsy. Pathology sections showed an inflammatory infiltrate of histiocytes, lymphocytes, and neutrophils. They also demonstrated some necrosis with surrounding gliosis.

The diagnosis of TDL was deemed the most probable given the clinical picture and the results of the workup. Further testing, notably for anti-MOG antibodies and CSF oligoclonal bands, was declined by the parents for financial reasons.

Treatment was initiated with intravenous pulse methylprednisolone at a dose of 500mg per day for five days and the patient had a remarkable recovery with complete resolution of her headache, diplopia, and both facial as well as upper limb weakness. There was some residual lower limb weakness which gradually resolved over the following four weeks. She was discharged home with outpatient physiotherapy.

At the three-month follow-up, the patient had no residual symptoms.

## Discussion

First described in 1979 in a patient with MS [6], TDLs were then conceptualized as being either an MS variant or a disease entity on a spectrum between MS and ADEM [7]. Today, TDLs have been reported in the literature associated with a variety of acquired CNS demyelinating diseases including MS and its atypical variants, ADEM, NMOSD, and MOGAD [1,4,8]. Their exact pathogenesis remains unclear.

Large-scale data on the natural history and epidemiology of TDLs is scarce but several cohort studies have reported on their frequency in MS, MOGAD, and NMOSD. TDLs can occur in up to 2% of MS patients, 5% of NMOSD patients, and over 20% of MOGAD patients [8,9]. The prevalence of “isolated” TDL, with no other underlying CNS demyelinating disease, is unknown. TDLs can occur at any age (2-73 years), with cases in the pediatric population, such as our patient, as well as in older adults [3-5].

As implied by their name, TDLs can mimic various space-occupying lesions, essentially tumors but also infectious processes such as abscesses. Consequently, they can be a cause of considerable diagnostic difficulty especially if the lesion is solitary and there is no known underlying demyelinating disease.

The clinical presentation is rarely discriminative as both TDLs and space-occupying lesions can have similar symptoms with headache, limb weakness, seizures, or cognitive impairment. TDLs tend to have an acute or subacute onset with gradual worsening [1,5]. Studies in the pediatric population have reported that children usually have a more markedly acute onset (<1 week) [3], a finding we did not observe in our patient whose disease course progressed over a month.

It is of utmost importance to distinguish between TDLs and space-occupying lesions in order to avoid unnecessary invasive diagnostic procedures when possible. Several radiological clues on conventional MRI have been reported. MRI features suggestive of tumefactive demyelination include (1) open-ring or incomplete rim enhancement with the incomplete portion usually abutting the gray matter,(2) lack of mass effect, and (3) relatively mild perilesional edema [1,5]. All three features were consistent with our case although some of the lesions had a complete ring of enhancement.

TDLs can be solitary or multiple and are usually supratentorial, with the frontal and parietal lobes being the most common sites [1,5]. The posterior fossa and basal ganglia can also be involved, both locations being suggestive of underlying MOGAD [8]. In rare cases, TDLs can also involve the spinal cord [10,11].

Unfortunately, conventional MRI cannot always reliably differentiate between tumefactive demyelination and space-occupying lesions, especially in the presence of a solitary lesion with less typical features (complete ring or patchy enhancement, or pronounced mass effect). In these cases, advanced imaging techniques such as MRI spectroscopy, MRI perfusion, or PET scan can be helpful, but are not always easily accessible, even more so in resource-limited settings as in our case. Furthermore, the results can be inconclusive as findings from TDLs and tumors sometimes overlap [1,5,12].

If a lumbar puncture can be performed, CSF shows slightly elevated protein levels and mild pleocytosis but can be completely normal [1,4,5,8], as was the case with our patient. Oligoclonal bands (OCB) are positive in about half of the patients with TDLs, some of which develop MS on follow-up. Generally, oligoclonal band positivity is less frequent in demyelinating disease with TDL-onset. It can also be a clue on the underlying demyelinating disease, OCB being much more suggestive of MS compared to MOGAD or NMOSD [8].

Given the current literature, testing for both anti-MOG and anti-AQP4 antibodies seems reasonable and particularly relevant in the context of tumefactive demyelination.

In difficult cases with inconclusive workup, brain biopsy is sometimes warranted and ultimately performed in order to exclude malignancy. Even then, pathology is not devoid of errors due to possible sampling bias (i.e.: insufficient or non-representative tissue, fixation quality), and experienced neuropathologists are needed to correctly interpret findings [5]. Pathology sections can show varying degrees of demyelination, macrophage infiltration, reactive astrocytes, and gliotic tissue along with perivascular lymphocytic cuffing [3,13].

There are no guidelines on the treatment of tumefactive demyelination. Based on data from cohorts and case series, first-line therapy involves high-dose corticosteroids followed by plasma exchange (PLEX) in the case of no or insufficient response, with intravenous immunoglobulins (IVIG) also being reported as a second-line therapy for some patients [1,4,5,14,15]. If first and second-line therapies fail, immunosuppressive treatment is indicated but there is no consensus on the choice and modalities of therapy [1,4,5,8]. In cases with underlying demyelinating conditions such as MS, NMOSD, or MOGAD, the treatment follows the standard therapy of the disease [5,14].

Prognosis is usually good: TDLs are characteristically steroid-responsive and have a mostly benign course. Mortality has rarely been reported, notably in brainstem localizations [5].

Regarding the follow-up of TDLs, the paucity of data on their natural history makes it difficult to predict their evolution to monophasic or recurrent disease. Highly heterogeneous recurrence rates have been reported in the literature, ranging from 10% to almost 70% [4,5,16].

The nosology of tumefactive demyelination is also still unclear: it could represent a distinct CNS demyelinating disease as postulated by Kepes et al. in 1993, a variant of MS, or another designation for atypical forms like Schilder’s or Marburg’s [7,17,18]. TDLs can also be associated, as mentioned previously, with other demyelinating diseases such as ADEM, MOGAD, or NMOSD [1,8].

Whether or not patients will develop one of the characterized CNS demyelinating conditions mentioned above is still debatable and warrants further research.

## Conclusions

Tumefactive demyelinating lesions are uncommon and can pose a considerable diagnostic challenge. They can be observed in isolation or associated with a wide variety of acquired demyelinating diseases such as MS, MOGAD and NMOSD.

Our case illustrates the overall lack of guidelines on the modalities of their diagnosis and treatment as well as the need for better recognition of new-onset tumefactive demyelination: how extensive does the workup need to be? When can the clinician confidently make the diagnosis without a biopsy? When does pathology become indispensable? Can we predict which patients will recur or convert to a well-defined acquired demyelinating disease such as MS? Further data is needed to decisively answer these questions and fill the lingering gaps of knowledge.

## References

[1] Nakayama M, Naganawa S, Ouyang M, Jones KA, Kim J, Capizzano AA, Moritani T (2021). A review of clinical and imaging findings in tumefactive demyelination. AJR Am J Roentgenol.

[2] Patriarca L, Torlone S, Ferrari F, Di Carmine C, Totaro R, di Cesare E, Splendiani A (2016). Is size an essential criterion to define tumefactive plaque? MR features and clinical correlation in multiple sclerosis. Neuroradiol J.

[3] McAdam LC, Blaser SI, Banwell BL (2002). Pediatric tumefactive demyelination: case series and review of the literature. Pediatr Neurol.

[4] Fereidan-Esfahani M, Decker PA, Weigand SD (2023). Defining the natural history of tumefactive demyelination: A retrospective cohort of 257 patients. Ann Clin Transl Neurol.

[5] Zhang Y, Zhang T, Zhang X (2023). Clinical spectrum and prognosis of pathologically confirmed atypical tumefactive demyelinating lesions. Sci Rep.

[6] van der Velden M, Bots GT, Endtz LJ (1979). Cranial CT in multiple sclerosis showing a mass effect. Surg Neurol.

[7] Kepes JJ (1993). Large focal tumor-like demyelinating lesions of the brain: intermediate entity between multiple sclerosis and acute disseminated encephalomyelitis? A study of 31 patients. Ann Neurol.

[8] Cacciaguerra L, Morris P, Tobin WO (2023). Tumefactive demyelination in MOG AB-associated disease, multiple sclerosis, and AQP-4-IgG-positive neuromyelitis optica spectrum disorder. Neurology.

[9] Fereidan-Esfahani M, Decker PA, Eckel Passow JE, Lucchinetti CF, Flanagan EP, Tobin WO (2022). Population-based incidence and clinico-radiological characteristics of tumefactive demyelination in Olmsted County, Minnesota, United States. Eur J Neurol.

[10] Pérez CA, Patnaik A, Oommen S, Redko A, Mathis SB (2020). Tumefactive demyelinating lesions in children: a rare case of conus medullaris involvement and systematic review of the literature. J Child Neurol.

[11] Brinar M, Rados M, Habek M, Poser CM (2006). Enlargement of the spinal cord: inflammation or neoplasm?. Clin Neurol Neurosurg.

[12] Takenaka S, Shinoda J, Asano Y (2011). Metabolic assessment of monofocal acute inflammatory demyelination using MR spectroscopy and (11)C-methionine-, (11)C-choline-, and (18)F-fluorodeoxyglucose-PET. Brain Tumor Pathol.

[13] VanLandingham M, Hanigan W, Vedanarayanan V, Fratkin J (2010). An uncommon illness with a rare presentation: neurosurgical management of ADEM with tumefactive demyelination in children. Childs Nerv Syst.

[14] Altintas A, Petek B, Isik N (2012). Clinical and radiological characteristics of tumefactive demyelinating lesions: follow-up study. Mult Scler.

[15] Weinshenker BG, O'Brien PC, Petterson TM (1999). A randomized trial of plasma exchange in acute central nervous system inflammatory demyelinating disease. Ann Neurol.

[16] Li X, Miao X, Wang Y (2022). Central nervous system tumefactive demyelinating lesions: Risk factors of relapse and follow-up observations. Front Immunol.

[17] Poser S, Lüer W, Bruhn H, Frahm J, Brück Y, Felgenhauer K (1992). Acute demyelinating disease. Classification and non-invasive diagnosis. Acta Neurol Scand.

[18] Weinshenker BG (2015). Tumefactive demyelinating lesions: Characteristics of individual lesions, individual patients, or a unique disease entity?. Mult Scler.

